# Facts Relative to Milk-Sickness

**Published:** 1840

**Authors:** Wm. J. Barbee

**Affiliations:** Late of Marshall, Illinois; Cincinnati


### Art. IV.—*Facts relative to the Endemic Disease called, by the people of the West, Milk-Sickness.* By Wm. J. Barbee, M. D., late of Marshall, Illinois.

My observations and experience upon this singular malady are quite limited. I am induced to contribute my mite towards solving its mystery, upon the principle that the aggregate of small items makes up the sum of human knowledge. I have no conjectures to offer, but wish to state a few facts which have come within my own knowledge, touching its *locality*, its *vegetable origin*, its *symptoms*, *treatment*, and *anatomical characters*.

*Of its locality.*—I have seen the seat of this endemic in Kentucky, Ohio, Indiana and Illinois.

In Kentucky it prevailed with some mortality, three and four years since, in Harrison county, a few miles from Cynthiana, on a small creek, chiefly amid rolling and hilly land. An account of it was published from the Inaugural Thesis of Doctor John Newton Smith, now of Paris, Ky., in the Transylvania Journal for April, 1837.

In Ohio, upon Mad river and the Great Miami, the destroyer has been making its victims for the last thirty years. I remember, seven years since, when a student of medicine, residing at Troy, on the Miami river, there were several cases, one of which I saw; and that they all occurred on a particular side of the stream, and so prevalent was the opinion that the disease was confined to one side, that the butchers would never purchase their beef from that quarter; and the inquiry was common at the market-house, "on what side of the river was this beef killed?"

In Indiana a few cases occurred, during the past summer and fall, in Vermillion, Vigo, Sullivan and Knox counties, all of which are situated on the Wabash river.

In Danville, ——— county, Indiana, during the fall of '38, and the succeeding winter, spring and summer, as many as sixty persons died of the disease, together with a number of the cattle.

In Illinois, several cases have occurred in Edgar county, about twenty miles northwest of the Wabash river.

All of these localities I have seen, with the exception of Danville, and I have never been able to discover in them any peculiarity as to topography or geology. Nor have any circumstances in the history of the "Milk Sick" in these districts enabled me to approach nearer to a discovery of its cause, than that it probably has a *vegetable origin.* What plant, flower, vine or root, out of all the rich vegetation of the west, can be the cause of so dire a malady as the sick stomach? Be it what it may, it seems to seek no peculiar station. It is on the rocky cliff and in the rich meadow; on the green hillock and in the gloomy swamp. It grows in the thick forest, and upon the wide-spread prairie.

By some it would be deemed altogether unnecessary to offer any thing in support of the supposition of the vegetable origin of this disorder. This is the most popular theory, and has more facts to sustain it than that which contends for a mineral poison; or another, perhaps the most fanciful, which boldly declares the disease to be owing to some peculiar modification of the atmospheric air—a theory applied to every obscure epidemic, and one which serves as a cloak for our ignorance.

But I am rather departing from my design. As long as the subject remains a matter of doubt, it becomes no one to affirm positively that the cause of the "sick stomach" is, or is not a vegetable, mineral or ærial poison; but, from all the information I possess, I am induced to believe that *several vegetable substances may give rise to the complaint.*

And here let me ask, why should we endeavor to limit the cause of any disease to a single agent? Why, for example, will authors tell us that *the cause* of intermittent fever is marsh miasm, when observation shows that atmospheric vicissitude is a cause (to mention no other) almost equally as powerful.

But to the facts.

Cows have been enclosed in large woodland pasture, or let out to range upon particular sections of land, without having access to any other water than that from a neighboring creek or branch, which, from common and constant use, was known not to contain any deleterious agent, and their milk has, in numbers of cases, given rise to the disease. The same cows have died of *the trembles.*

In November, 1838, a family of six persons, travelling westward, put up at a house a few miles east of Terre Haute, Indiana. At breakfast they all partook freely of *butter* and *milk*, and immediately departed on their journey. By the time they reached Illinois, in five or six hours, they were all taken with nausea, vomiting, &c. and died, every one of them, in from two to six days. Upon inquiry it was ascertained that the place where they had taken their breakfast was in a "milk-sick" region.

About the same time I saw, in Terre Haute, Doct. David Dale Owen, then State Geologist of Indiana, who informed me that he had been, for a day or two previous, in a "milk-sick" district in the northern part of Vigo county, and that a plant was given him by a farmer of that section of country, which he believed was the cause of the complaint. This plant, we decided, was the *Eupatorium ageratoides*, although we might have been mistaken, as we both acknowledged ourselves poor botanists.

Dr. Owen stated, that he had made a decoction of the leaves and stem, and administered a draught of it to a calf. In a short time it had what is called "*the trembles;*" became subsequently parlysed, stiff in its joints, uttered a most doleful noise, and in a few hours died.

In Vincennes, Knox county, Indiana, during the past fall, a number of citizens were under considerable alarm, in consequence of the death of a few persons in the neighborhood, who had been taken soon after eating *butter* and drinking *milk*, obtained from cows who died with the trembles. At the same time several cattle, who had been feeding on the opposite side of the river, were found dead. A number of families of the town were afraid to use any butter brought from the country. This information I obtained from a respectable citizen of that place. I believe a professional gentleman of Vincennes had, at that time, some dried specimens of a plant which was supposed to be the cause of the disease.

I have conversed with physicians residing in Sullivan county, Indiana, and in some of the eastern counties of Illinois, and, from all the evidence I could collect, I was induced to believe that the disease had its origin in some poisonous vegetable.

In addition to the plant already mentioned, I have been shown specimens of the *R. Radicans*, poison vine; *R. Toxicodendron*, poison oak; and *R. Vernix*, swamp sumach; and, from several gentlemen of Indiana and Illinois, I have been assured that their cattle have been affected like the calf destroyed by the Eupat. ageratoid., in consequence, as they believed, of feeding upon these several vegetables.

I have given the Eupat. ageratoid. and the R. Toxicodendron to dogs, and the effects upon both animals seemed nearly similar. I am sorry that I cannot here give an exact detail of these two experiments. I noted them down accurately at the time, and placed the paper with several others which I have not with me. I think, however, I can state the result in general from memory.

1*st Dog.*—Took the Eupat. ager. in decoction; had no effect for an hour, when he began to shake by intermittent jerks, as dogs usually do when they are said to have the "shaking palsy;" in half an hour after this, vomiting commenced; matter ejected of a dark appearance and very offensive smell; in a short time he became prostrated, whirled about on the ground, until, finally, he lost the power of motion, became stiff, tried to howl, and in about three hours from taking the decoction he was dead.

2*d Dog*—Took a decoction of R. *Toxicodendron.* Its effects were manifested in half an hour—he ran round the yard, at first very playfully, but soon commenced yelling as if he had been injured or scared. Occasionally he would fall down, and falling would make a long stagger. In about an hour from the commencement of the experiment vomiting commenced, accompanied by violent spasms—the animal drew himself up as if the misery he was suffering were in the abdomen. The pupil was almost closed. Respiration finally became very hurried and laborious, and in two hours from taking the draught he died.

Now what do these experiments prove? The *probability* that the dogs had what, among cows, is denominated "*the trembles*," and that the affection may be produced by more than one plant.

There are a great number of noxious vegetables growing every where in the west which are as fatal to life as the poison causing the "milk sickness." Who that has lived in Ohio or Kentucky has not seen cows lying in rows under buck-eye trees? I once assisted in the dissection of a cow found in this situation, and upon opening her stomach and bowels they were found literally loaded with half-chewed buck-eyes. Yet a cow may be turned out in buck-eye woods and fed with impunity; she may either eat the buck-eyes or not, and in case of eating them, the quantity taken may not be sufficient to destroy life; or her vital powers may resist the poisonous agency of any quantity. This fact, I am confident, obtains in the disease under consideration—a cow may feed in a pasture where the "milk-sick" season exists. She may never touch it, or she may eat or drink freely of it and continue in good health. Another cow, perhaps, feeding with her will contract the trembles and either recover or die. This remark may be extended to other inferior animals, and to the human subject. Individuals, as well as several of the lower animals possess different susceptibilities to the disease. Horses and hogs, it would seem, are not so liable to the disease as cows and dogs. And, of a given number of individuals who have partaken alike of poisoned *butter* and *milk*, some will merely sicken, others will vomit, and others will die, the whole with or without treatment. I would respectfully offer these facts in reply to the remark of one of the editors of the Journal, that the sick stomach could not be owing to the R. *Radicans* or *Eupat. agerat.*, inasmuch as these plants grow throughout the west, while the disease is "*perseveringly limited to certain localities.*"

There are certain dietetic and therapeutic articles which, when received into the stomach, have different destinations and exert different influences; increasing the activity of the brain, of the heart, or of the secretion of bile, urine, &c.—and further, these substances may affect the quality of secretions as well as the quantity. We have for example a number of articles which exert a change upon the chemical constitution of the urine. In the same way the milk of the cow may be altered in its quality by several agents taken into the stomach, so as to change its nutritive virtue into a poison.

*Symptoms.*—Most writers upon this disease divide it into three stages:—1. The forming stage. 2. The stage of excitement and vomiting, and 3. The stage of collapse. All of these may or may not be well marked. Circumstances may so operate upon a case as to blend the different stages together or to change their character, as here laid out, in such a manner that no one can readily distinguish them. For convenience, however, the division may be admitted. I derive all that I have to say upon the symptoms from two cases which recently came under my observation, regretting that my experience is so limited.

Mr. F——, a traveller, had been riding through a "milk-sick" region and treated the matter as a humbug, declaring that he was not afraid to eat any butter or drink any milk that might be placed before him. I was called to him about eleven o'clock at night—he was attacked at dark, and according to the description of the bystanders complained at first of giddiness and weakness, became stupid and indisposed to move. In the course of an hour he said he was burning up at the stomach, and shortly afterwards vomiting commenced. This continued till near the time of my arrival, when he was so completely exhausted that he could not speak. I could scarcely feel his pulse—the extremities were cold, and his breathing scarcely audible. In about an hour from the time that I reached the house he died.

Mr. D—— was taken on September 14th, 1839, with general debility amounting to an overwhelming feeling of depression and smothering—inability and disinclination to move—loss of appetite and listlessness, accompanied by a peculiar fœtor of the breath, which cannot be described. To me it was not unlike the smell of a small pox patient. In a few hours the patient complained of nausea and a burning sensation at the stomach, which was soon followed by vomiting. Shortly after this occurred I saw him and learned from conversation with his wife, that he had on that day killed a young heifer, and cooked a piece of the meat on the coals and eat it, and that no other person had tasted it. I expected to find the vital energies greatly prostrated, but an examination of the pulse agreeably disappointed me. This was about 90, pretty full and strong, but I was afraid to employ venesection. The pupils were dilated but a strong light contracted them. The head was quite hot, and likewise the body—but the extremities were slightly cold. The patient lay semi-comatose, and I felt confident that any powerful depleting course of treatment would but hasten the sinking stage which was fast approaching. Nothing had passed the bowels for twenty-four hours, and I should have mentioned that before my arrival he had complained several times of severe spasm of the bowels.

The management of this case, together with some reflections, will constitute what I have to say on the subject of

TREATMENT.

In treating this case I was guided by first principles and facts. The symptoms were such as to convince me that *gastritis* was present, accompanied by spasmodic action of the bowels, more particularly of the colon in its descending portion; and further that there was a strong tendency to intense susception in this bowel. This condition of the colon I regarded as a chief cause of the obstinate constipation always present in the disease; and as the experience of practitioners coincided in establishing the strong probability of a patient's safety in the event of free evacuations, I directed my attention particularly to this point. But in effecting this important end, I was cautious of the means employed. I have noticed with no little surprise that many western physicians have declared (and some of them in medical journals,) that nothing but drastic purgatives will save a "milk-sick" patient. This is their logic: the bowels are locked up—the worst possible form of constipation exists—purgation must be effected—mild laxatives will not answer the purpose—therefore something active must be employed. Now let all the facts with regard to this pathological state of the bowels be admitted. I think the deduction is a wrong one which leads to the administration of drastic purges. The strong tendency to gastritis, if indeed this does not already exist, forbids the use of any thing so irritating. I would as soon think of giving brandy to allay the action of the heart and arteries—or a large dose of calomel, jalap and gamboge to cure a dysentery. But there is another consideration which should induce us to withhold any thing of a very stimulating character to the stomach and bowels, as well as to be guarded in adopting an active antiphlogistic course of treatment. We have something more than a simple gastritis to contend with—we have a gastritis the result of a *narcotic irritant;* we have, as will be more clearly shown hereafter, a constricted, if not a spasmodic condition of the colon; and last, but not least, we have a brain and nervous system, in truth the whole system, laboring under the influence of a poisonous agent. While then we are directing our efforts to restore to healthy action the stomach and bowels, believing that if these be corrected the other organs will take care of themselves, let it ever be borne in mind that the whole man is sick from head to foot, and that as every constitution has its peculiarities our treatment must be modified accordingly.

To proceed to the treatment of the case just mentioned. I applied six cups to the region of the stomach, and ordered cold affusion to the head. Sponging the body with vinegar and water, and bathing the feet and legs in hot water. In the course of an hour, the vomiting abated considerably, and in order to palliate his thirst, I gave him a few soda powders. Having thus composed his stomach, reduced the force and frequency of the pulse and the heat of the head, and established a more equal circulation—I left him with a mild cathartic of calomel and rhubarb, and milk-warm slippery-elm tea as a drink—giving special directions to keep his head cool and his feet warm. In five hours I returned. Cathartic had had no effect upon the bowels—patient had complained several times of severe spasms. Head was pretty cool, feet warm; gave him a dose of castor-oil and left him again—returned in five hours—no evacuation, some burning of the stomach—pulse eighty; occasionally spasms of the bowels which appeared to be confined to the left side of the abdomen and to take the course of the descending colon. I at once determined to put into execution a measure which I had resolved upon previously, in the event of continued constipation. I introduced into the rectum a flexible gum elastic tube, about eighteen inches long, and passed it up into the colon nearly the whole length; into this I succeeded in injecting about a quart of warm salt and water. In less than fifteen minutes the patient had a large fæcal evacuation, at first compacted, but gradually assuming the consistence of mush, and a bilious colour. This was succeeded in the course of an hour by a second evacuation of the same character, and I left him with directions to take in a couple of hours ten grains of blue mass. On the next day he had a good appetite and felt disposed to leave his bed. This, however, I prohibited, and by watching him closely for two or three days succeeded in restoring him to health.

*Anatomical characters,*—The calf which was destroyed by the Eupat. ageratoid. was examined a short time after death by Dr. Owen, and the only pathological condition he mentioned to me was a dry, contracted and thickened appearance of the coats of the stomach.

The following is the autopsy of the two dogs:

1st Dog, who took the Eupat. ageratoid. Lining of the mouth, fauces and œsophagus fiery red.

*Stomach*—size smaller than natural; in every direction corrugated. Hypertrophy of muscular and mucous coats—mucous coat inflamed; presented distinct patches of permanent vermillion redness; some softening in the spots not affected with hyperæmia.

*Duodenum.*—Mucous lining of superior part, red.

*Jejunum and Ileum*—natural.

*Colon.*—Descending portion contracted, so that the greatest diameter which could be found did not exceed three quarters of an inch, and it presented the appearance of being bound by ligatures in several places.

*Peritoneum*—natural.

*Liver* was withered and wrinkled, almost exsanguineous, and all the bile seemed to have collected in the gall bladder, which was very much distended,

*Thoracic viscera.*—Very black blood in the parenchyma of the lungs; rest healthy.

*Brain* was not examined.

2d Dog, to whom the R. Toxicodendron was administered. The same pathological states were discovered, with the exception of an intussusception in the colon in place of the several constrictions.

*Autopsy of Mr. F.*

External appearance, emaciated; physiognomy frightful—looked like an old man of eighty.

*Cavity of the abdomen.* Stomach—muscular coat natural; colon—puckered and so contracted as to shorten the longitudinal diameter; cardiac and pyloric orifices rigid; mucous coat deep red and thickened in spots, balance presenting a rather pale and softened appearance.

*Duodenum, Jejunum and Ileum* in their peritoneal covering inflamed.

*Colon.*—Descending portion contracted to near the size of a common candle; two very narrow places, as in the gut of the first dog—and about two inches above the sigmoid flexure there was a partial intussusception.

*Liver* shrunk; quite blue; gall bladder distended.

*Spleen and Kidneys* natural.

*Thorax.*—Mucous coat of bronchiæ and air-cells very red.

*Lungs* contained considerable black blood.

*Heart and Pleura* natural.

Permission could not be obtained to examine the brain.

A word or two and I shall close this paper. I deem my knowledge of this dreadful malady very trifling; and as I, with hundreds of others, wish to acquire some information respecting it, it is to be hoped that all who have had practice in the disease, will give the profession the result of their observation and experience.

*Cincinnati,* 1840.

